# Comparative Analysis of Olive-Derived Phenolic Compounds’ Pro-Melanogenesis Effects on B16F10 Cells and Epidermal Human Melanocytes

**DOI:** 10.3390/ijms25084479

**Published:** 2024-04-19

**Authors:** Juhee Cho, Meriem Bejaoui, Kenichi Tominaga, Hiroko Isoda

**Affiliations:** 1 Alliance for Research on the Mediterranean and North Africa (ARENA), University of Tsukuba, Tsukuba 305-0006, Japan; cho.juhee.gf@un.tsukuba.ac.jp (J.C.);; 2 Open Innovation Laboratory for Food and Medicinal Resource Engineering, National Institute of Advanced Industrial Science and Technology (AIST), Tsukuba 305-8577, Japan; 3 Faculty of Life and Environmental Sciences, University of Tsukuba, Tsukuba 305-8577, Japan

**Keywords:** olive-derived natural compound, glutaraldehyde compound, melanogenesis, B16F10 cells, human epidermal melanocyte

## Abstract

Olive leaf contains plenty of phenolic compounds, among which oleuropein (OP) is the main component and belongs to the group of secoiridoids. Additionally, phenolic compounds such as oleocanthal (OL) and oleacein (OC), which share a structural similarity with OP and two aldehyde groups, are also present in olive leaves. These compounds have been studied for several health benefits, such as anti-cancer and antioxidant effects. However, their impact on the skin remains unknown. Therefore, this study aims to compare the effects of these three compounds on melanogenesis using B16F10 cells and human epidermal cells. Thousands of gene expressions were measured by global gene expression profiling with B16F10 cells. We found that glutaraldehyde compounds derived from olive leaves have a potential effect on the activation of the melanogenesis pathway and inducing differentiation in B16F10 cells. Accordingly, the pro-melanogenesis effect was investigated by means of melanin quantification, mRNA, and protein expression using human epidermal melanocytes (HEM). This study suggests that secoiridoid and its derivates have an impact on skin protection by promoting melanin production in both human and mouse cell lines.

## 1. Introduction

The skin, which accounts for 15% of the body weight, is the largest organ in the human body [1]. The skin plays an extremely important role in maintaining homeostasis, such as temperature and water content, and providing a physical barrier against mechanical injuries, chemical insults, microbial infection, and radiation [1,2,3]. Melanogenesis acts as an especially unique defense system against ultraviolet (UV) radiation by producing melanin pigment [3]. Melanin is a crucial determinant of skin color and is produced in melanocytes located in the basal layer of the epidermis [4,5,6]. Our skin is constantly exposed to harmful chemicals that cause DNA damage, oxidative stress, and inflammation, mainly through sunlight [7,8,9]. To protect the skin against DNA damage from UV radiation, melanogenesis is triggered [10,11]. Briefly, melanin pigment is produced in melanocytes and then stored within specialized membrane-bound organelles termed melanosomes [12,13]. Melanocytes extend their dendrites to facilitate the transfer of melanosomes efficiently to adjacent keratinocytes [14]. Tips of melanocyte dendrites come into contact with 30–40 surrounding keratinocytes, forming a functional unit called a melanin unit that allows keratinocytes to distribute melanin throughout the skin properly [15,16,17,18]. This process is regulated by numerous signaling pathways, like the cAMP pathway, MAPK pathway, and WNT/β-catenin pathway [19,20]. Melanocortin 1 receptor (MC1R) stimulates melanogenesis and pigmentation by binding with alpha-melanocyte-stimulating hormone (α-MSH) and adrenocorticotropic hormone (ACTH), its physiological agonists, and activated MC1R induces an increase in cAMP levels and photoprotective eumelanin synthesis [21,22,23]. The mitogen-activated protein kinase (MAPK) signaling pathway is also one of the major signaling cascades regulating cellular processes, including cell proliferation, differentiation, and apoptosis [24]. The interaction of stem cell factor (SCF) and its receptor, c-Kit, triggers the activation of MAPK family members, including p38, ERK, and JNK [25,26,27,28]. In addition, the Wnt/β-catenin signaling pathway directly induces the differentiation of melanocyte stem cells into melanocytes and contributes to melanogenesis [29]. Wnt ligand binds to the receptor Frizzled, a G-protein-coupled receptor, leading to inhibition of glycogen synthase kinase-3 beta (GSK3β) and accumulation of β-catenin [29,30,31,32]. Subsequently, these pathways converge to microphthalmia-associated transcription factor (MITF) and induce MITF and melanogenic enzymes’ expression [19,20,33]. Those melanogenic enzymes, tyrosinase (TYR), tyrosinase-related protein 1 (TYRP1), and DOPAchrome tautomerase (DCT), catalyze the biosynthesis steps of eumelanin or pheomelanin [34,35]. However, endogenous melanin is not enough to protect our skin. Generally, the inherent sun protection factor (SPF) of fair human skin ranges from two to four, and melanin is able to absorb only 50–75% of UV radiation [36,37,38]. Furthermore, dysfunction in those melanogenesis pathways leads to pigmentary disorders on the skin, such as vitiligo and postinflammatory hypopigmentation [39,40]. Hence, it is necessary to develop drugs without side effects for the purpose of evening out the skin tone, minimizing UV damage, and aesthetic reasons. In this context, natural compounds can be considered promising sources in cosmetics and pharmaceuticals for skin integrity. Olive also contains a variety of natural compounds that have beneficial health effects [41].

Olive, including olive leaves and olive oil, has been predominantly consumed as a traditional diet and utilized in folk medicine in Mediterranean countries [42,43]. Numerous studies have reported the beneficial effects of the Mediterranean diet on various chronic diseases, including cardiovascular diseases, cancer, and diabetes. As a result, there has been a worldwide increase in interest in and consumption of olive products [44,45]. Olive contains great amounts of polyphenols, such as oleuropein (OP) and hydroxytyrosol. However, the constituents of these polyphenols can vary depending on several factors, including olive varieties, extraction techniques, and environmental conditions [46]. Nevertheless, some compounds are commonly found in olive fruits and leaves, including OP, one of the abundant phenolic compounds in olive oil and leaves [47]. It belongs to a specific group called secoiridoids. These secoiridoid compounds from olive are a potential candidate for pharmaceutical applications due to their health properties. Recent studies have reported their high biological activity, such as anti-inflammatory, anti-cancer, and neuroprotective effects [48]. Notably, it is worth mentioning two other types of phenolic compounds which are highly found in olives, namely, oleocanthal (OL) and oleacein (OC). However, not all species contain OL and OC. Miho et al. [49] explored 80 olive cultivars and reported the variability of phenolic composition derived from the different cultivars. Due to the fact that some olive species do not naturally contain OL or OC, there are limitations in their application. However, it is worth noting that OL and OC can be synthesized from OP due to their structural similarity [50]. This synthetic approach provides an alternative source of OL and OC, overcoming the limitations of their natural occurrence in certain olive species. Thus, in this current study, we succeeded in developing a new methodology for synthesizing OC from OP with a high yield. While the conventional method requires more than 10 steps, our study enables the one-step synthesis of OC using solid acid catalysts [51]. This development paves the way for the large-scale application of OC.

Despite the many health benefits of olives, the specific effects of the individual olive component on the skin have yet to be studied intensively. Nevertheless, recent studies have reported the anti-inflammatory effect and UV protection effects of olive extract [52,53,54]. Building on those findings, in this study, we aimed to screen the effects of three olive-derived compounds (OP, OL, and OC) on melanin production through mouse melanoma B16F10 cells, which originate from melanocytes and human epidermal melanocytes. We achieved this by conducting global gene expression profiling using B16F10 cells and subsequently validating the effects on HEM cells.

## 2. Results

### 2.1. Upregulation of Mitf and Melanogenic Enzymes in B16F10 Cells

The B16F10 mouse melanoma cell line is well known for its melanin production capability and serves as a convenient tool for preliminary study. In this study, B16F10 cells were utilized for the initial screening of OP, OL, and OC. First, the cytotoxic effects of OP, OL, and OC against murine melanoma cells were evaluated. Those olive-derived phenolic compounds exhibited non-cytotoxic effects, as depicted in Figure S1. Therefore, the lowest concentration of 5 μM was chosen for further study.

**Figure 1:**
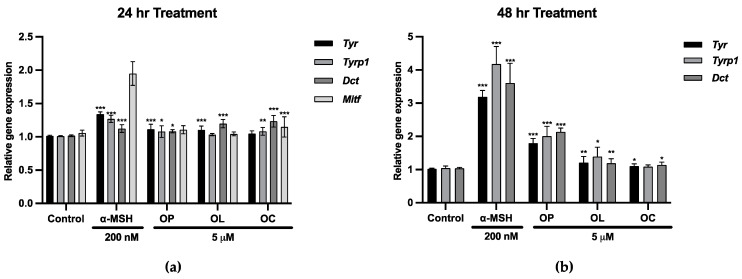
The gene expression of transcription factor *Mitf* and melanogenic enzymes was measured. The mRNA level was quantified using TaqMan real-time PCR. B16F10 cells were treated with 5 μM of olive-derived phenolic compounds for 24 and 48 h. (**a**) Gene expression of *Mitf*, *Tyr*, *Tyrp1*, and *Dct* after 24 h of treatment. (**b**) Gene expression of *Tyr*, *Tyrp1*, and *Dct* after 48 h of treatment. The results of three independent experiments are expressed as the mean ± standard deviation (SD). * *p* < 0.05, ** *p* < 0.01, and *** *p* < 0.001.

It is reported that olive extract and one of its main components, OP, have effects on pro-melanogenesis as well as UV protection effects [55,56,57]. To investigate the effects of olive-derived secoiridoids (OP, OL, and OC), we identified MITF and melanogenic enzymes’ expression on B16F10 cells. MITF transcriptionally regulates those enzymes (TYR, TYRP1, and DCT) known to be involved in each step of melanin synthesis [58]. Despite the enhanced expressions of these markers, the melanin production induced by OP, OL, and OC was less pronounced compared to the positive control, α-MSH (Figure 1). Nonetheless, their potential to stimulate melanogenesis in B16F10 cells is evident.

### 2.2. Impact of OL and OC on Global Gene Expression in B16F10 Cells

To understand the underlying molecular mechanisms and biological effects of olive-derived phenolic compounds, we conducted global gene expression profiling, focusing primarily on OL and OC. This experimental design was informed by the extensive research already conducted on OP, prompting us to explore the lesser-studied effects of OL and OC. Such an approach allows us to contribute novel insights into the broader impacts of these compounds.

**Figure 2:**
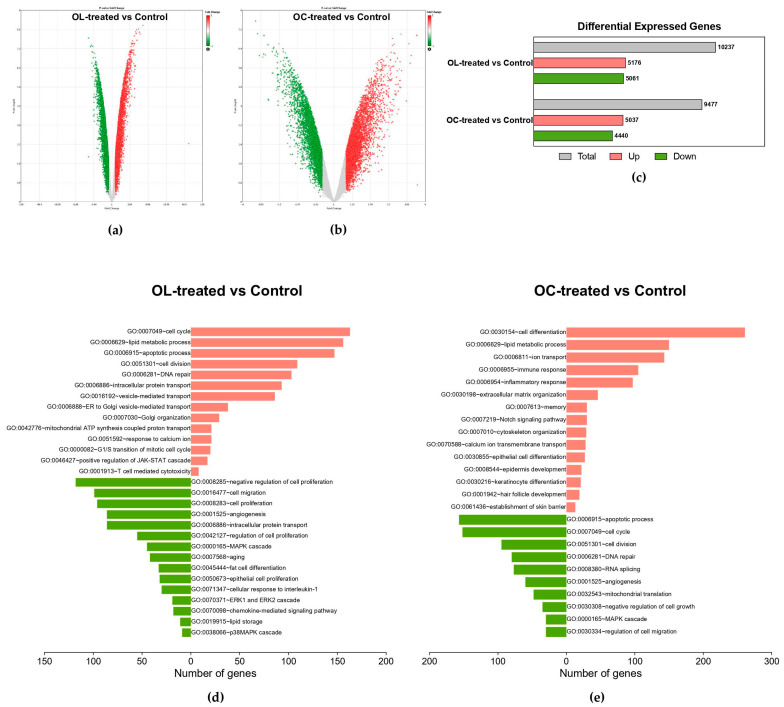
Characteristics of gene expression profiling in OL- and OC-treated B16F10. Volcano plots exhibiting the DEGs regulated by (**a**) OL-treated vs. control and (**b**) OC-treated vs. control. The *y*-axis displays the log_10_ (*p*-value), and the *x*-axis represents the fold change. Up- and downregulated DEGs are presented as red and green dots, respectively. (**c**) Bar graph showing the number of DEGs regulated by 5 μM OL and OC. (**d**) Significantly enriched gene ontology biological process (GOBP) by up- and downregulated DEGs in 5 μM OL-treated B16F10. (**e**) Significantly enriched gene ontology biological process (GOBP) by up- and downregulated DEGs in OC-treated B16F10.

Microarray analysis was performed using RNA samples from B16F10 cells treated with the control (untreated cells), the positive control (200 nM α-MSH ), and 5 μM OL and OC. The differentially expressed genes (DEGs) meeting the criteria of a fold change (FC) of ≥1.2 or ≤−1.2 were selected for the analysis. The FC values of total gene probe sets after treatment with OL or OC compared to the control are displayed as volcano plots in Figure 2a,b. Of the OL-treated vs. control (untreated cell), 10,237 genes were differentially expressed, of which 5176 DEGs were upregulated and 5061 DEGs were downregulated. In the case of 5 μM OC vs. control, 9477 genes were differentially expressed, of which 5037 DEGs were upregulated and 4440 genes were downregulated (Figure 2c).

**Figure 3:**
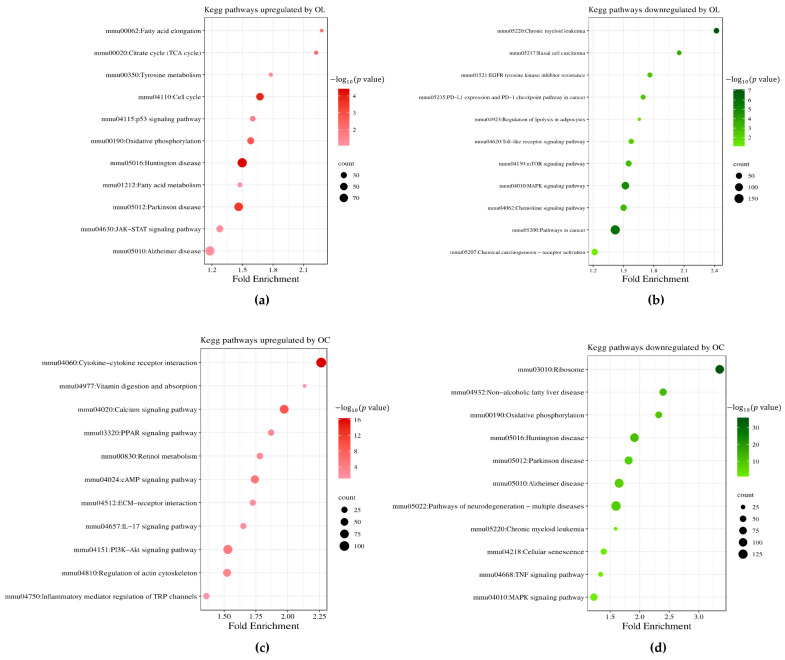
Significantly up- and downregulated KEGG pathways by OL- and OC-treated B16F10. (**a**) Up- and (**b**) downregulated KEGG pathways by OL treatment. (**c**) Up- and (**d**) downregulated KEGG pathways by OC treatment. The *x*-axis corresponds to fold enrichment, and the *y*-axis shows the terms of signaling pathways. The color code represents a negative log-transformed *p* value, and the bubble size represents the number of DEGs in each pathway.

For further investigation of the regulated biological processes and pathways, we used the Database for Annotation, Visualization, and Integrated Discovery (DAVID). Figure 2d shows a significantly enriched gene ontology biological process (GOBP) by OL-treated cells. GOBP terms, including cell cycle (GO:0007049), apoptotic process (GO:0006915), response to calcium ion (GO:0051592), and positive regulation of JAK-STAT cascade (GO:0046427), were significantly affected by the upregulated DEGs of OL-treated B16F10 compared to control. On the other hand, DEGs downregulated by OL treatment significantly affected the GOBPs related to cell migration (GO:0016477), cell proliferation (GO:0008283), angiogenesis (GO:0001525), and MAPK cascade (GO:0000165). Furthermore, we explored the KEGG pathways regulated by OL treatment. The top up- and downregulated KEGG pathways, along with their corresponding negative log-transformed *p* value and the numbers of DEGs, are presented in Figure 3a,b. We found that the cell cycle, tyrosine metabolism, p53 signaling pathway, and several neuron-related pathways (Huntington’s disease, Parkinson’s disease, Alzheimer’s disease) were upregulated by OT (Figure 3a). In contrast, the mTOR signaling pathway, MAPK signaling pathway, and pathways in cancer were downregulated by OL treatment (Figure 3b).

Although those two molecules, OL and OC, have similar structures, they showed different bioactivities. Interestingly, the significantly upregulated DEGs by OC treatment enriched the GOBP in terms of cell differentiation (GO:0030154), notch signaling pathway (GO:0007219), epidermis development (GO:0008544), and keratinocyte differentiation (GO:0030216) (Figure 2e). Also, downregulated DEGs of OC treatment affected the BP terms, including the apoptotic process (GO:0006915), cell cycle (GO:0007049), angiogenesis (GO:0001525), and MAPK cascade (GO:0000165) (Figure 2e). In addition, we found that OC downregulated the pathways related to neuronal diseases, including Huntington’s disease, Parkinson’s disease, and Alzheimer’s disease, as opposed to OL (Figure 3d). On the contrary, OC upregulated the calcium-signaling pathway, cAMP-signaling pathway, and PI3K-Akt-signaling pathway, related to keratinocyte differentiation and melanogenesis (Figure 3c).

Our microarray analysis revealed distinct bioactivities between OL and OC despite their similar structures. OL treatment significantly upregulated genes involved in the cell cycle, tyrosine metabolism, and various neuron-related pathways while downregulating those in the mTOR and MAPK signaling pathways and pathways related to cancer. In contrast, OC primarily influenced gene expression related to cell differentiation, epidermis development, and melanogenesis-associated pathways like the PI3K-Akt signaling pathway. These differential gene expressions between OL and OC highlight their unique molecular mechanisms and potential therapeutic applications in cellular processes and neurodegenerative diseases.

### 2.3. Hierarchical Clustering of DEGs of Enriched Functions in OL- and OC-Treated B16F10 Cells

**Figure 4:**
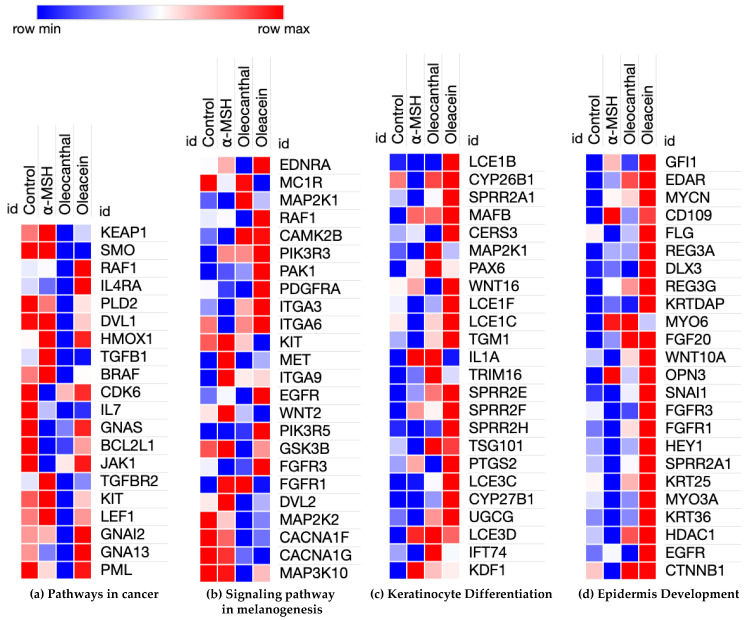
Heatmap representing relative gene expression by OL and OC on B16F10 cells. (**a**) Anti-cancer and (**b**) melanogenesis-related genes were downregulated compared to control and α-MSH. (**c**,**d**) show that OL and OC upregulated the genes related to skin homeostasis for protection.

To investigate the individual gene expression of GO and the pathways found above, we deeply probed the relative expression and gene function through hierarchical clustering. OL and OC were compared using hierarchical clustering. The open-source software Morpheus was used to generate a heat map (https://software.broadinstitute.org/morpheus/ accessed on 18 December 2023). The results revealed that OL and OC have different bioactivities (Figure 4a–c). Figure 4a shows that OL and OC regulate genes involved in cancer pathways such as the MAPK/ERK pathway (BRAF, RAF1), immune-related pathways (IL4RA, IL7, JAK1), and the cell cycle (CDK6) [59,60,61]. These major oncogenes were remarkably decreased by OL treatment compared to OC. Additionally, Figure 4b exhibits the relative gene expression of the melanogenesis pathway. Melanogenesis and melanoma share common pathways. Several components of the MAPK pathway (MAP2K1, RAF1), the PI3K/AKT pathway (PIK3R3, PIK3R5), and the Wnt pathway (WNT2, GSK3β) are stimulated by OL and OC treatment. On the contrary, OC markedly upregulated the genes related to keratinocyte differentiation and epidermis development. The UGCG, keratin (KRT), late cornified envelope (LCE), and small proline-rich protein (SPRR) families are essential factors for keratinocyte differentiation (Figure 4c,d). Differentiated keratinocytes replenish the upper layer and desquamate the dead cells to renew the skin [62]. These results suggest that the olive-derived phenolic compounds might inhibit melanoma growth by disrupting pathways and the cell cycle in melanoma, and might provide the underlying molecular mechanism of the anti-cancer effects of OL and OC reported in previous studies [63,64,65,66]. Interestingly, OL and OC highly upregulated the keratinocyte-related genes that specifically regulate keratinocyte differentiation and epidermis development. These results imply that OL and OC also have potential as skin protection agents.

### 2.4. Validation of Melanogenesis and Keratinocyte Differentiation-Associated Genes Regulated by OL and OC

To validate the microarray results regarding skin-related effects, melanogenesis, and keratinocyte differentiation, we utilized RT-PCR to evaluate the gene expression of the significantly regulated DEGs upon treatment with OL and OC. Here, OP was chosen as a comparative reference to OL and OC, since it has been reported recently that OP has effects on pro-melanogenesis, UV protection, and skin protection [55,56,57].

**Figure 5:**
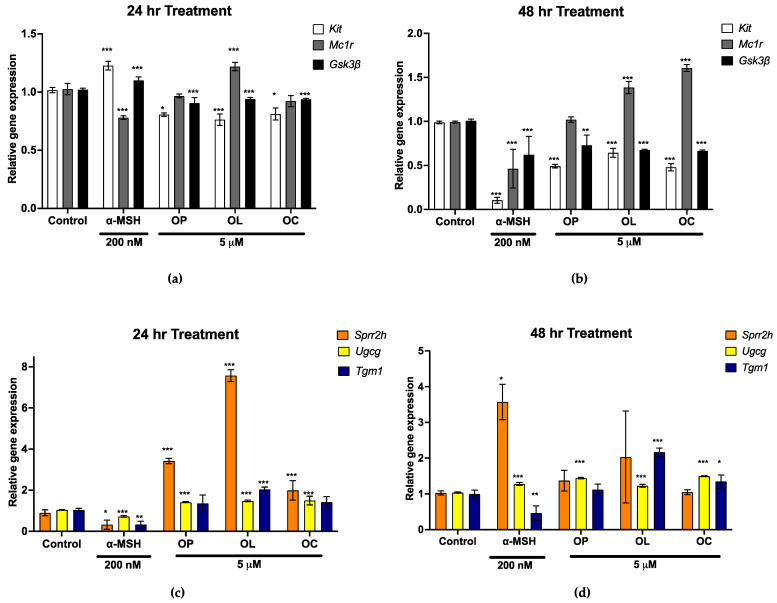
Gene expression of melanogenesis and keratinocyte terminal differentiation marker. To validate the microarray analysis, we evaluated the gene expressions of several significant markers and quantified them as relative gene expressions compared to control (untreated) in B16F10 cells. Melanogenesis-related gene expression, *Mc1r*, *Gsk3β*, and *Kit* were investigated on B16F10 cells after (**a**) 24 h and (**b**) 48 h of treatment. Keratinocyte terminal differentiation markers and *Sprr2h*, *Ugcg*, and *Tgm1* expression were quantified after (**c**) 24 h and (**d**) 48 h of treatment. The results of three independent experiments are expressed as the mean ± SD. * *p* < 0.05, ** *p* < 0.01, and *** *p* < 0.001.

Through comprehensive global gene expression profiling, we selected significantly regulated melanogenic genes by OL and OC. *Mc1r* and receptor tyrosine kinase (*Kit*) are the key genes that stimulate the cAMP and MAPK signaling pathways in melanogenesis [67]. Gsk3β is the downstream gene of the Wnt and PI3K/AKT pathway [68]. Thus, we quantified the gene expression of these markers on B16F10 cells treated for 24 and 48 h. In the first 24 h, OP and OC seemed to downregulate *Mc1r* expression as microarray data, while OL upregulated *Mc1r* expression (Figure 5a). However, olive-derived secoiridoids remarkably upregulated *Mc1r* expression after 48 h of treatment. *Gsk3β* and *Kit* expression levels were downregulated after 24 and 48 h of treatment (Figure 5a,b). These results show that OL and OC play a role in promoting melanin synthesis.

Noteworthily, OC significantly enhanced genes associated with epidermis development and keratinocyte differentiation. Hence, critical markers of keratinocyte terminal differentiation (*Sprr2h*, *Ugcg*, and *Tgm1*) were selected from the enriched genes through microarray analysis. The gene expression levels of *Sprr2h*, *Ugcg*, and *Tgm1* were markedly elevated by OP, OL, and OC after 24 h and 48 h of treatment on B16F10 cells (Figure 5c,d). These results suggest that OL and OC exhibit potential effects on both melanogenesis and keratinocyte differentiation.

### 2.5. Effect of the Olive-Derived Phenolic Compounds on Melanin Production in Human Epidermal Melanocyte (HEM)

**Figure 6:**
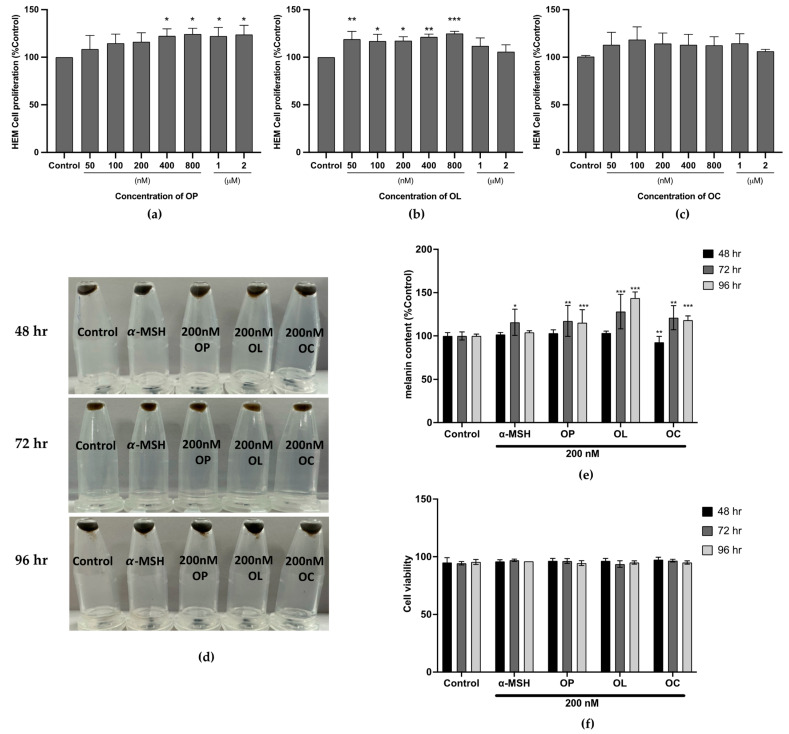
The cell cytotoxicity of olive-derived compounds and melanin content in HEM was evaluated. HEM cells were treated with (**a**) OP, (**b**) OL, and (**c**) OC for 48 h in a dose-dependent manner. (**d**–**f**) Melanin contents in HEM treated with OP, OL, and OC were investigated in a time-dependent manner (48, 72, and 96 h). (**d**) Pellets were obtained after treatment. (**e**,**f**) Relative melanin contents compared to control and cell viability were quantified in HEM cells treated with 200 nM of OP, OL, and OC in a time-dependent manner. The results of three independent experiments are expressed as the mean ± SD. * *p* < 0.05, ** *p* < 0.01, and *** *p* < 0.001.

While B16F10 cells provide a valuable model for preliminary study, it is still necessary to validate our findings in primary human melanocytes that closely represent the genuine human skin environment. Accordingly, human epidermal melanocytes (HEM) were used for further investigation. First, the cytotoxic effects of OP, OL, and OC against HEM were assessed. Cells were treated in lower doses from 50 nM to 2 μM of olive-derived compounds for 48 h, and the cell proliferation rate was measured using the MTT assay. Figure 6a shows that OP increased the proliferation rate as the concentration increased, especially notably in the range of 400 nM to 2 μM, where significant effects were observed without cytotoxicity. For OL, significant changes were detected in the range of 50–800 nM (Figure 6b). In this concentration range, cell proliferation was enhanced without inducing cytotoxicity. As shown in Figure 6c, OC did not exhibit significant changes in proliferation rates across the tested concentration range. However, the proliferation rate at all tested concentrations was higher than the control (non-treated). For subsequent studies, a lower concentration, 200 nM, was selected.

Furthermore, the melanin contents in HEM treated with those phenolic compounds were measured in a time-dependent manner. The results revealed that OP, OL, and OC increased the melanin content, as the color of the pellet became darker after treatment (Figure 6d). The pellet sizes were increased, and the color became darker as the treatment time increased. These effects were quantified compared to the control, and the results indicate that the melanin contents began to be enhanced after 72 h of treatment. Compared to control, melanin treated with OP, OL, and OC for 72 h increased up to 117.33%, 128.14%, and 121.10% in the cases of OP, OL, and OC, respectively (Figure 6e). After 96 h, the melanin contents were changed to 115.3%, 143.8%, and 118.3%. Interestingly, this effect was achieved without compromising cell viability, and the increase in melanin content was more pronounced compared to the effect observed with the positive control, α-MSH (115.76% (72 h) and 104.1% (96 h)) (Figure 6f).

### 2.6. Expression of Transcription Factor MITF and Melanogenic Enzymes Were Upregulated in HEM Cells

Prior to discussing our findings, it is crucial to address the variations in treatment durations employed in our study. We carefully designed our treatment durations to align with the distinct phases of melanocyte biology, ensuring accurate observation of both the gene and protein expression dynamics relevant to melanin synthesis. For the melanin assay, durations of 48, 72, and 96 h were chosen to accommodate the gradual process of melanin synthesis in melanocytes, which necessitates a longer treatment for the activation and functioning of key enzymes like TYR, TYRP1, and DCT. Conversely, the gene expression analysis was conducted over shorter treatment periods of 24 and 48 h, since *MITF*, an upstream gene, exhibits changes earlier than the melanogenic enzymes. Furthermore, we extended the treatment time to 48 and 72 h for protein expression analysis, aligning with the time required for the translation of RNA into functional proteins.

**Figure 7:**
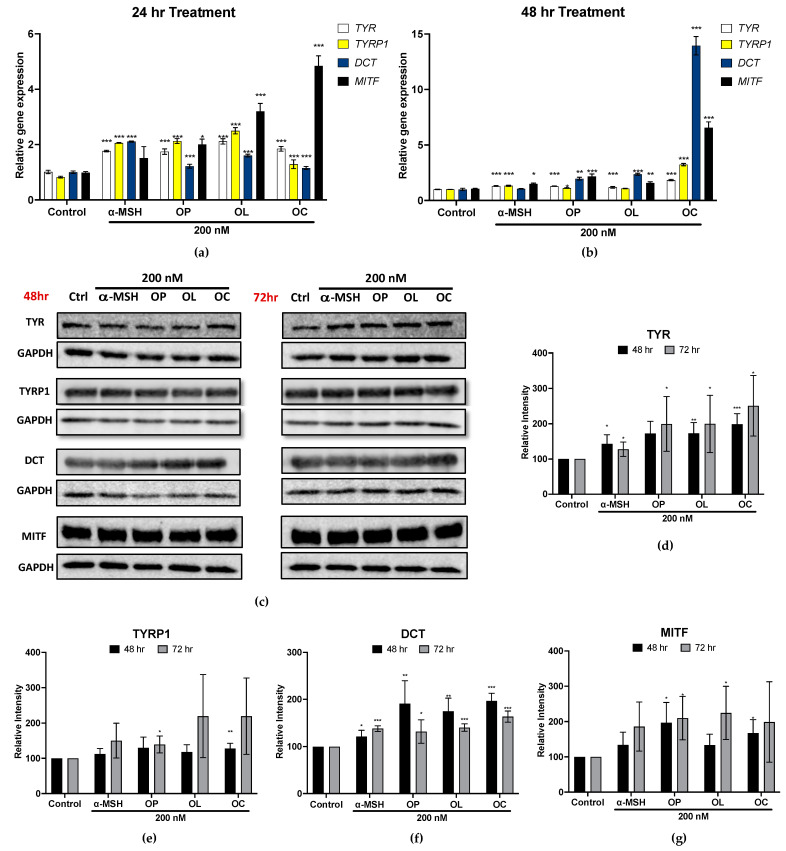
Gene and protein expression in HEM cells treated with OP, OL, and OC in a time-dependent manner. (**a**,**b**) Gene expression of *MITF* and melanogenic enzymes in HEM cells after 24 and 48 h of treatment. (**c**) Protein bands were obtained from HEM treated with OP, OL, and OC for 48 and 72 h (**d**–**g**). The relative band intensities of MITF, TYR, TYRP1, and DCT were compared to GAPDH using the LI-COR system. The results of three independent experiments are expressed as the mean ± SD. * *p* < 0.05, ** *p* < 0.01, and *** *p* < 0.001.

Following this premise, gene and protein expression of MITF and melanogenic enzymes were investigated in human melanocytes. Figure 7a,b show the gene expression on HEM cells treated with OP, OL, and OC for 24 and 48 h. After 24 h, olive-derived compounds significantly enhanced *MITF* and the expression of its downstream genes (*TYR*, *TYRP1*, *DCT*). In particular, OP and OL demonstrated a significant upregulation of *TYR* and *TYRP1* expression compared to α-MSH after 24 h. Meanwhile, OC exhibited a notable upregulation of melanogenic gene expression, including *MITF*, *TYR*, *TYRP1*, and *DCT*, after 48 h of treatment, surpassing the effects observed with α-MSH and other compounds.

Furthermore, protein bands of MITF, TYR, TYRP1, and DCT were obtained in HEM after 48 and 72 h, and the band quantification was assessed using GAPDH as a loading control. The results demonstrated that all samples upregulated the protein expression of the melanin-producing enzymes (TYR, TYRP1, and DCT) after 48 and 72 h (Figure 7c,d). MITF expression was also increased by OP, OL, and OC treatment. However, its expression decreased after 72 h, while other melanogenic enzymes increased. Presumably, MITF activated melanogenic enzymes during the initial 48 h of treatment, after which it started to decrease. The results consistently showed an increasing tendency of mRNA and protein expression. Altogether, OP, OL, and OC effectively regulated the proteins involved in melanin production.

### 2.7. Elucidation of Molecular Mechanisms through Validation of Downstream Markers

**Figure 8:**
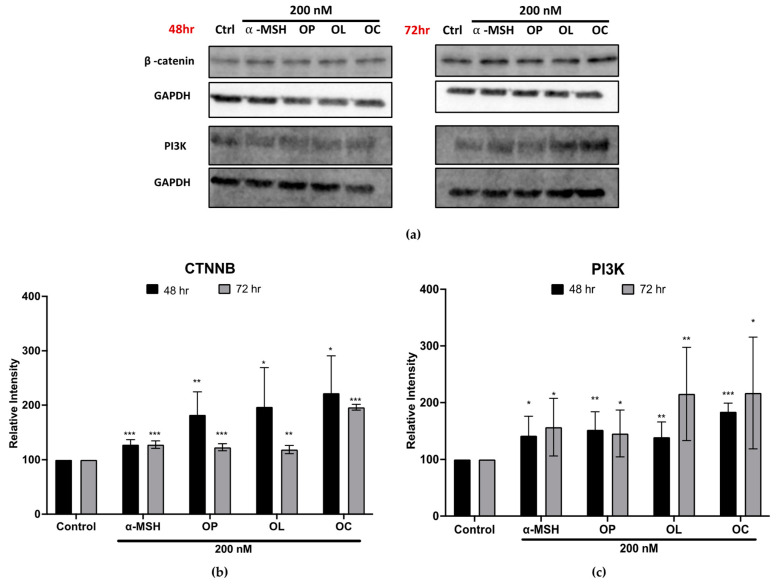
Protein expression in HEM cells treated with OP, OL, and OC in a time-dependent manner. (**a**) Protein bands were obtained from HEM treated with OP, OL, and OC for 48 and 72 h. (**b**,**c**) The relative band intensities of β-catenin and PI3K were compared to GAPDH using the LI-COR system. The results of three independent experiments are expressed as the mean ± SD. * *p* < 0.05, ** *p* < 0.01, and *** *p* < 0.001.

In continuation of our investigation into melanin production regulation, the molecular mechanism influenced by olive-derived compounds was explored. Based on our microarray analysis, the compounds were found to be effective in upregulating markers related to epidermal differentiation, development, and skin development, particularly pertinent to melanocytes. We further observed the downregulation of *Kit* and *Gsk3β* on B16F10 cells, indicative of the melanogenic potential. This suggests their regulatory roles in melanocyte development and Wnt pathway modulation [29,35]. Expanding our findings to HEM cells, the expression of downstream markers, PI3K and β-catenin, was probed. Figure 8 shows the bands detected from HEM cells treated with OP, OL, and OC. Following 48 h of treatment, the relative band intensity of β-catenin exhibited an increase in response to OP, OL, and OC. Notably, the band intensity surpassed the positive control, α-MSH. However, with prolonged treatment to 72 h, β-catenin expression was slightly decreased compared to 48 h. In addition, PI3K expression also increased after both 48 and 72 h of treatment, demonstrating a time-dependent upregulation.

The results, as shown in Figure 7, revealed the activation of MITF and key melanogenic enzymes in response to olive-derived compounds. Additionally, the data presented in Figure 5 indicate a potential regulatory effect on membrane receptors, specifically Kit and MC1R, which play crucial roles in melanogenesis. This comprehensive validation of downstream markers in both Wnt and PI3K signaling pathways provides deeper insights into the underlying molecular mechanisms of the melanogenesis pathway.

### 2.8. Molecular Interaction of MC1R with Olive-Derived Compounds

**Figure 9:**
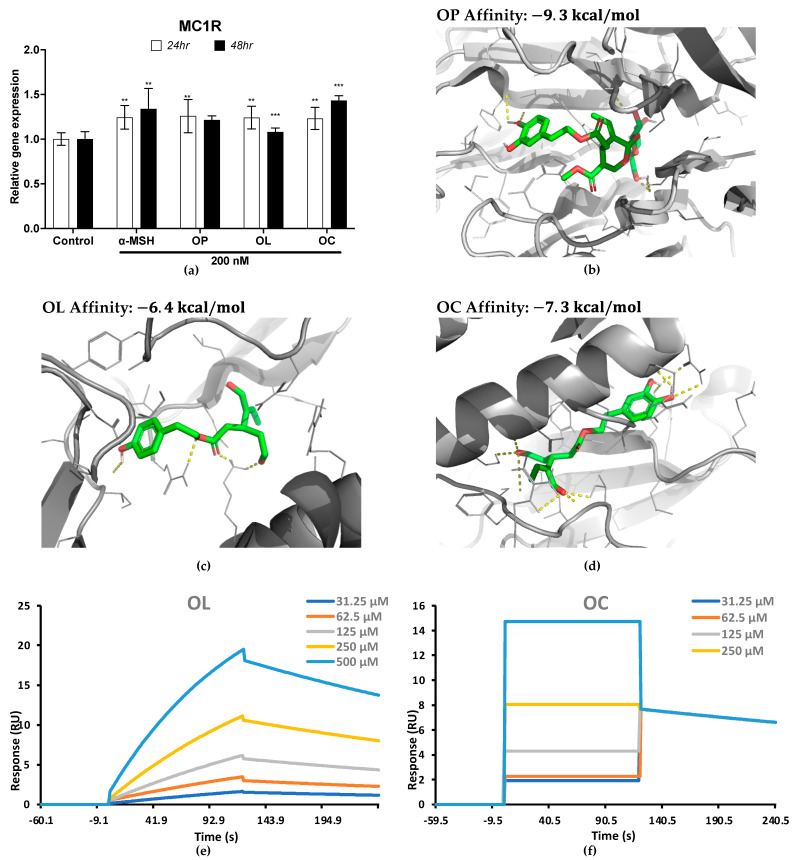
Gene expression in HEM cells and docking simulation on MC1R and olive-derived compounds. (**a**) MC1R expression in HEM treated with OP, OL, and OC in a time-dependent manner. (**b**–**d**) Predicted MC1R complex bound with OP, OL, and OC by molecular docking simulation. (**e**) Kinetic analysis on OL. (**f**) Kinetic analysis on OC. The results of three independent experiments are expressed as the mean ± SD. * *p* < 0.05, ** *p* < 0.01, and *** *p* < 0.001.

Intrigued by the observed upregulation of *Mc1r* expression in B16F10 cells following exposure to olive-derived compounds, we sought to unravel the underlying molecular mechanisms of MC1R regulation that are crucial for transducing signals into cells [23]. We validated MC1R expression in HEM cells. The results revealed a substantial upregulation of *MC1R* expression by OP, OL, and OC, underscoring the potential roles of these compounds in modulating MC1R activity (Figure 9a).

Employing a multi-faceted approach, we initiated molecular investigations by simulating the interaction between MC1R and olive-derived compounds through molecular docking simulations using AutoDock Vina. These simulations not only provided predictive insights into the binding affinity and potential binding model of these compounds with the MC1R receptor, but also laid the foundation for subsequent experimental validation. The results showed the binding with the lowest affinity with the most favorable interaction between the MC1R protein and the ligands (Figure 9b–d). In the docking simulation, OP bound to the MC1R protein with a binding affinity of −9.3 kcal/mol. Compared to OL and OC, which had binding affinities of −6.4 kcal/mol and −7.3 kcal/mol, respectively, OP seemed to form the strongest binding with MC1R.

To substantiate the in silico predictions, we conducted a surface plasmon resonance (SPR) analysis (Figure 9e,f and Table 1). Through SPR analysis, we were able to obtain the association (ka) and dissociation constant (kd), as well as the equilibrium dissociation constant (KD) (Table 1). The results showed that OC (3.515 × 10^−9^) had a lower KD value compared to OL (1.925 × 10^−4^). This suggests that MC1R complex-bound OC has a stronger interaction and more stability than OL. On the other hand, in our SPR analysis on OP, no significant response units (RU) were observed, indicating an absence of detectable binding under the experimental conditions employed. This could be attributed to a potentially low affinity of OP for the target.

Our research presents a comparative analysis of the interactions between olive-derived compounds (OP, OL, and OC) and the MC1R receptor, utilizing both molecular docking simulations and surface plasmon resonance (SPR) analysis. While molecular docking suggested OP as having the strongest binding affinity to MC1R, SPR analysis revealed no significant binding for OP, highlighting a notable divergence between in silico predictions and experimental outcomes. Conversely, OC demonstrated a stronger and more stable interaction with MC1R in SPR analysis compared to OL, despite showing lower affinity in docking simulations. These findings emphasize the intricate nature of ligand–receptor interactions and the critical need for integrating multiple analytical approaches to unravel the full spectrum of molecular dynamics and binding behaviors in biological systems.

## 3. Discussion

The skin plays a vital role in providing a physical barrier between organisms and the environment and preventing the invasion of pathogens and chemical and physical assaults [69]. Melanogenesis is one of the homeostatic processes that protects epidermal keratinocytes and dermal fibroblasts from UV damage [70]. Melanocytes that have the ability to produce melanin are derived from the neural crest [71,72]. These neural crest-derived melanocytes are differentiated and trigger pigmentation in the epidermis [73,74]. This melanocyte development is induced by numerous transcriptional and signaling regulations, such as SCF/KIT pathway and master regulator MITF, and is also associated with melanin production [75,76,77]. Nonetheless, the melanin content without any photoprotective agents is insufficient to protect skin completely from DNA damage under repeated UV exposure (especially in the summer) [36,37]. Thus, there is a strong demand for good photo-protectors that can enhance the natural cutaneous pigmentation in the skin. In this context, olive extract and its major components were studied for their effects on melanogenesis and photoprotection [52,53,54]. However, previously, the effects of OL and OC on the skin were barely known. Therefore, we proposed screening the skin-associated effects of OL and OC by means of global gene expression analysis using B16F10 cells and investigating the pro-melanogenesis effect on HEM cells.

In this study, we utilized the B16F10 murine melanoma cell line as a primary in vitro model to investigate the effects of olive-derived phenolic compounds on melanogenesis. This choice was driven by the cell line’s well-documented consistency in melanin production [78,79,80,81]. However, we acknowledge the limitations inherent in using a non-human melanoma cell line, particularly when translating findings to normal human melanocytes. While B16F10 cells are an effective tool for preliminary studies in cancer and melanogenesis, differences in species biology may impact the direct applicability of these results to human conditions. This limitation was carefully considered in our research design and interpretation of results.

Moreover, in our microarray analysis with B16F10 cells, we observed that OL and OC not only influenced melanogenesis, but also downregulated genes related to cancer progression, implicating these compounds in the dual roles of melanin regulation and potential cancer therapeutics. It is crucial to note, however, that the mechanisms underlying melanogenesis and cancer progression, while overlapping, can differ significantly between murine and human systems. Therefore, we emphasize caution in extrapolating these findings to human melanoma without further validation.

We also included human epidermal melanocytes (HEM) in our study to provide a more direct insight into human skin biology. The use of both murine and human cell lines in our research reflects a balance between the need for reliable models for preliminary screening (B16F10) and subsequent validation in a human-specific context (HEM). This complementary approach, despite its inherent limitations, contributes to a more comprehensive understanding of the effects of olive-derived phenolic compounds on melanogenesis and, potentially, melanoma progression.

In the preliminary screening using B16F10 cells, the expression of MITF and its downstream genes was confirmed first. The results revealed that olive-derived phenolic compounds moderately increased *Tyr* and *Dct* expression. Subsequently, global gene expression analysis was performed as a preliminary study to screen the effects of OL and OC, especially on the skin, and to elucidate their molecular mechanisms on melanogenesis. As a consequence, we confirmed that DEGs related to cancer and melanogenesis were downregulated by OL and OC. These biological processes are deeply related and share common signaling pathways. It is widely known that melanin protects the skin from UV [82]. On the other hand, activation of melanogenesis in a cancer cell can rather enhance melanoma progression [83,84]. In our results, OL and OC downregulated pivotal target genes in melanoma therapies. The mTOR and Ras/Raf/ERK/MEK (MAPK) are major pathways in cancer therapeutics and are commonly activated by mutation [85,86,87]. Dysregulation of those pathways contributes to carcinogenesis, apoptosis, invasion, and metastasis [87,88]. OL and OC downregulated the *RAF1*, *BRAF*, *MTOR*, *AKT1*, and *MAPK* families in B16F10 cells. Also, the *FGF* families of the FGF-FGFR pathway and *KEAP1* of the KEAP1-Nrf2 pathway, which acts on tumor growth and chemo-/radiation-resistance, were downregulated by these two glutaraldehyde-like compounds [89,90,91,92]. Downregulation of these pathways suppresses cancer and inhibits melanin production. It is most likely that the oncogenes in melanoma cells confer to effectively suppress tumors, and their mutation restricts melanin synthesis through olive-derived phenolic compounds. Nevertheless, the gene expressions performed to validate microarray analysis showed that *Mc1r* was increased after 48 h of treatment and *Gsk3β* was decreased by OL and OC. Furthermore, *Tyr* and *Dct*, which are melanogenic enzymes participating in melanin biosynthesis, were upregulated. These results showed that olive-derived glutaraldehyde compounds may have a potential effect on regulating melanin production.

Remarkably, we found additional skin-related biological processes from global gene expression analysis. OC significantly upregulated skin development, epidermal cell differentiation, epithelial cell development, and keratinocyte differentiation on B16F10 cells. OL also upregulated these factors, but the effect was not as dramatic as that of OC. Upregulation of those biological processes supports barrier formation in the skin. The epidermal keratinocytes of the basal layer are gradually differentiated and migrate toward the surface of the skin during this process [93]. Terminally differentiated keratinocytes result in the formation of cornified layers that provide a physical barrier to external stresses [94,95]. IVL, FLG, LOR, LCE, and SPRR genes are the structural elements of the cornified layer [96]. All these genes were upregulated in our microarray data. After the validation of gene expressions that showed high fold-change, we confirmed that *Sprr2h*, *Ugcg*, and *Tmg1* were increased after the treatment with OP, OL, and OC. These results suggest that this study is worthy, in that it reveals that OL and OC exert skin protection effects through upregulation of the elements of the cornified layer.

Based on these results from mouse melanoma cells, we investigated the melanogenesis effect of olive-derived compounds using human melanocytes to provide a more accurate representation of human biology [97]. We found that olive-derived secoiridoids activated melanin production more than α-MSH upon an increase in treatment time. Furthermore, the gene and protein expression of MITF, TYR, TYRP1, and DCT were increased after the treatment. Importantly, this finding provides evidence that olive-derived secoiridoids trigger melanin synthesis and can be supported by various studies on pro-melanogenesis [58,98,99]. Consequently, our results suggest that the binding of olive-derived secoiridoids to MC1R not only alters receptor activity, but also triggers downstream signaling pathways, leading to an upregulation of melanogenic enzymes and an increase in melanin production. This finding underscores the potential of olive-derived secoiridoids as active agents in modulating skin pigmentation processes, and it opens avenues for exploring their use in dermatological therapeutics and cosmetics. Additionally, building upon our current findings, future research could extend to co-culture systems involving dermal and epidermal cells, such as keratinocytes and fibroblasts. This would offer valuable insights into the physiological relevance of these interactions in a human skin mimic environment. These advanced approaches aim to further elucidate the molecular mechanisms at play and to more accurately evaluate the biological processes in a context that closely resembles human skin.

## 4. Materials and Methods

### 4.1. Sample Reagent Preparation

Oleuropein (≥98.0%, 12247) and oleocanthal (≥90.0%, PHL83882) were purchased from Sigma-Aldrich (St. Louis, MO, USA) (12247 and PHL83882). Pure Oleacein (≥97%) was synthesized according to a previous report [30] and provided by FoodMedOIL of AIST (Tsukuba, Japan). The samples were dissolved in 70% ethanol and diluted in the medium for use.

### 4.2. Cells and Cell Culture

The B16F10 mouse melanoma cell line and human epidermal melanocytes (HEM) were used. B16F10 cells were obtained from RIKEN (Tsukuba, Japan) and cultured in Roswell Park Memorial Institute (RPMI 1640) growth medium (Gibco, San Francisco, CA, USA) supplemented with fetal bovine serum (FBS, Gibco, USA) and 0.1% penicillin/streptomycin (Gibco, San Francisco, CA, USA).

HEM cells were purchased from Cell Applications and cultured in melanocyte growth medium: all-in-one ready-to-use (Cell Applications, Inc., San Diego, CA, USA). The cells were isolated from normal, healthy human neonatal foreskin.

The medium was changed every other day, and it was passaged when it reached 80–90% confluency using TrypLE Express (Gibco, San Francisco, CA, USA) and a Subculture Reagent Kit (HBSS, Trypsin/EDTA, and Trypsin Neutralizing Solution, Cell Applications, Inc., San Diego, CA, USA) each. The cells were kept under sterile conditions at 37 °C in a 75 cm^2^ flask (BD Falcon, Becton Dickinson, Oxford, UK) in a humidified atmosphere of 5% CO_2_. The cell viability was determined by trypan blue exclusion using a Countess 3 automated cell counter (Thermo Fisher Scientific, Waltham, MA, USA).

### 4.3. Cell Proliferation Assay

The MTT assay was used to determine the cytotoxicity of OP, OL, and OC. Briefly, B16F10 cells and HEM were seeded in 96-well plates (5 × 10^3^ cells/100 μL well and 1 × 10^4^ cells/100 μL well) and incubated overnight at 37 °C in a humidified atmosphere of 5% CO_2_. Then, B16F10 cells were treated with different concentrations of OP, OL, and OC from 5 μM to 40 μM. On HEM cells, which exhibit a lower proliferation rate and greater sensitivity to their microenvironment and stressors compared to B16F10 cells, we tested lower concentrations ranging from 50 nM to 2 μM [100]. After 48 h of treatment, 10 μL of 3-(4,5-dimethylthiazol-2-yl)-2,5-diphenyltetrazolium bromide (5 mg/mL MTT solution) was added to each well and further incubated for 4–8 h. Following that, 100 μL of 10% sodium dodecyl sulfate (SDS) was added and incubated overnight. The absorbance was measured at 570 nm using a microplate reader (Varioskan^TM^ LUX, Thermo Fisher Scientific, USA).

### 4.4. RNA Extraction

To extract the sample RNAs, B16F10 cells and HEM were seeded on 6-well plates (5 × 10^4^ cells/mL). Cells were incubated overnight and treated with α-MSH (positive control), OP, OL, and OC at 50–60% confluency. For B16F10 cells, 5 μM was used, and for HEM, 200 nM was used for treatment. RNA samples were extracted using ISOGEN II (Nippon Gene, Tokyo, Japan) from B16F10 cells and HEM following the manufacturer’s manual. Briefly, 1 mL of ISOGEN II was added after the treatment and incubated for 5 min at room temperature. Then, it was collected gently in a 1.5 mL tube, and 400 μL of distilled water was added. After incubating those tubes for 10 min at room temperature, the tubes were centrifuged at 12,000× *g* for 15 min. The supernatant was transferred to another tube and the same volume of isopropanol was added. After 10 min at room temperature, tubes were centrifuged at 12,000× *g* for 10 min. Lastly, the supernatant was removed and 700 μL of 75% ethanol was added. Before the use, ethanol was removed and RNA samples were dissolved in 20 μL of distilled water. The quality of RNA samples was measured using NanoDrop™ One/One^C^ Microvolume UV-Vis Spectrophotometer (Thermo Fisher Scientific, USA).

### 4.5. DNA Microarray Analysis

Template RNA samples were prepared from B16F10 cells as described above. After measuring the quality of the RNA samples, extracted RNAs (250 ng total RNA per sample) were amplified and biotinylated following the user’s guide. Next, cRNA samples were synthesized using an IVT PLUS Reagent Kit (Affymetrix Inc., Santa Clara, CA, USA). After purification of cDNAs, they were fragmented and labeled for hybridization, and 90 μL of cRNA samples were injected into the cartridge array and incubated for 16 h at 45 °C in a hybridization oven. The arrays were washed and stained with the GeneChipTM Fluidics Station 450 (Applied Biosystems™, Waltham, MA, USA), and imaging was conducted with a GeneChip^TM^ Scanner 3000 (Applied Biosystems™, USA). The transcriptome analysis console (TAC) version 4.0.1 was used to normalize the results.

### 4.6. Quantitative Real-Time PCR Analysis

The RNA samples were extracted as mentioned above. Extracted RNA was synthesized to cDNA using the SuperScript VILO IV (Thermo Fisher Scientific, USA) and quantified with a NanoDrop™ One/One^C^ Microvolume UV-Vis Spectrophotometer (Thermo Fisher Scientific, USA). Quantitative real-time PCR (qRT-PCR) was performed with a 7500 Fast Real-Time PCR (Applied Biosystems, USA) with TaqMan Master Mix and TaqMan probes and the following thermal cycling protocol: 95 °C for 10 min, 45 cycles of 95 °C for 15 s, and 60 °C for 1 min. The skin-related target genes were chosen based on the microarray results: Tyr (Mm00495817_m1), Tyrp1 (Mm00453201_m1), Dct (Mm01225584_m1), Mitf (Mm00434954_m1), Gsk3b (Mm00444911_m1), Kit (Mm00445212_m1), Mc1r (Mm00434851_s1), Sprr2h (Mm00488435_s1), Ugcg (Mm00495925_m1), and Tgm1 (Mm00498375_m1). The target genes for HEM were as follows: TYR (Hs00165976_m1), TRP1 (Hs00167051_m1), DCT (Hs01098278_m1), MITF (Hs01117294_m1), and MC1R (Hs00267167_s1). Gapdh (Mm99999915_g1) and GAPDH (Hs02786624_g1) housekeeping genes were used to normalize the relative gene expression.

### 4.7. Melanin Quantification

HEM cells were seeded onto 100 mm dishes at a concentration of 5 × 10^4^ cells/mL per dish and incubated in the same conditions as described above. Cells were treated at 60–70% confluency and incubated for 48, 72, and 96 h each. Then, the medium was removed and washed with HBSS (Cell Applications, Inc., USA). To harvest the cells, Trypsin/EDTA and Trypsin Neutralizing Solution (Cell Applications, Inc., San Diego, CA, USA) were used. Harvested cells were dissolved using 0.1% Triton X-100. The synthesized melanin was purified with 10% trichloro acetate and dissolved using 8N NaOH. Then, the melanin in 8N NaOH was heated for 2 h at 80 °C. The absorbance of the solution was measured at 410 nm, and the melanin content was calculated considering the cell viability and the number of cells.

### 4.8. Western Blot

Firstly, cells were seeded on 60 mm dishes at a concentration of 5 × 10^4^ cells/mL overnight. Afterwards, we treated HEM with 200 nM of OP, OL, and OC in a time-dependent manner (48–96 h). The protein samples were extracted using a mixture of radioimmunoprecipitation assay (RIPA) buffer (Sigma Aldrich, USA) and protease inhibitor (Sigma Aldrich, USA), following the manufacturer’s instructions. Then, the protein concentration was determined using a BCA Protein Assay (Thermo Fisher Scientific, USA). A sample of 20 μg of total protein was loaded onto 10% sodium dodecyl sulfate (SDS) polyacrylamide gel and transferred to a PVDF membrane (Millipore, Bedford, MA, USA) via electrophoresis (SDS-PAGE). The membrane was blocked in the Intercept^®^ (PBS) Blocking Buffer (LI-COR, Lincoln, NE, USA) for 2 h at RT. The protein expressions of MITF and melanogenic enzymes (TYR, TYRP1, and DCT) were detected by Western blot analysis using the following antibodies: recombinant anti-tyrosinase antibody (ab170905, Abcam, Boston, MA, USA), recombinant anti-TRP1 antibody (ab235447, Abcam, USA), anti-TRP2/DCT antibody (ab74073, Abcam, USA), anti-MiTF antibody (ab13703 Abcam and HPA003259 ATLAS ANTIBOIDES, USA), PI3 kinase antibody (#4292, Cell Signaling Technology, Danvers, MA, USA), and anti-beta catenin (ab6302, Abcam, USA) at 1/1000 dilution. Anti-GAPDH (ab9483, Abcam, USA) was used to normalize the protein loading variability. The membrane was blotted with these primary antibodies overnight at 4 °C and immersed in the secondary antibody, IRDye^®^ 800CW Goat anti-Rabbit or IRDye^®^ 800CW Goat anti-Mouse (LI-COR, USA). The signal was detected using the Odyssey Infrared Imaging System.

### 4.9. In Silico Molecular Docking

Computational protein–ligand docking analysis was performed using AutoDock Vina. The target protein, MC1R, was obtained from the protein data bank with ID: 7F4D. Ligand structures were obtained from the public chemical database PubChem. Autodock automatically found the predicted binding sites.

### 4.10. Surface Plasmon Resonance (SPR) Analysis

SPR analysis was performed using Biacore X100 (Cytiva, Uppsala, Sweden). The buffer was prepared with HBS-EP+ Buffer 10× (Cytiva, Sweden). MC1R human recombinant protein (Abnova, Taipei, Taiwan) was immobilized on a CM5 sensor chip (Cytiva, Sweden) using an amine-coupling method. Briefly, the surface was activated with EDC and NHS, and diluted MC1R in acetate pH 4.0 (1:1) was injected for 7 min. Afterward, unreacted sites were blocked with ethanolamine. The equilibrium dissociation constants (KD) between MC1R and OP, OL, and OC (500, 250, 125, 62.5 and 31.25 μM) were measured using Biacore X100.

### 4.11. Statistical Analysis

Microarray analysis was repeated twice. Other experiments were performed in three biological repetitions. All statistical tests were performed using GraphPad Prism 10.0.0 software for Mac. Student’s *t*-test or one-way analysis of variance (ANOVA) was used to compare the treatment condition with the control. Significance levels were represented as follows: * = *p* ≤ 0.05, ** = *p* ≤ 0.01, and *** = *p* ≤ 0.001.

## 5. Conclusions

**Figure 10:**
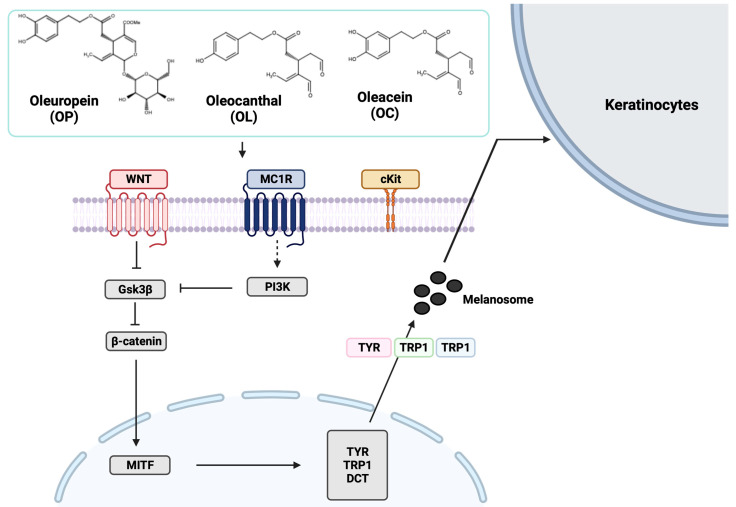
Scheme showing the mechanism of olive-derived secoiridoids that regulate melanogenesis signaling pathway. This figure was created with BioRender.com.

In summary, our findings suggest that olive-derived compounds promote melanin production through the activation of melanogenesis in human epidermal melanocytes. Furthermore, based on the results of genome transcriptomics analysis by microarray, we delved into the target genes on melanogenesis regulated by OL and OC. This study provides an estimated molecular mechanism of secoridoids from olive as a scheme shown in Figure 10. This study proposes a promising aspect of olive-derived secoiridoids for skin therapeutics and cosmetics. Future studies could investigate the effects on human skin mimics to clearly elucidate the mechanisms.

## Figures and Tables

**Table 1:** The kinetic association (ka) and dissociation constant (kd) of OL and OC were determined through kinetic analysis.

Kinetics Model	Target	Analyte	$\mathrm{Kinetics}\;Chi^{2}$ ($RU^{2}$)	ka (1/Ms)	kd (1/s)	KD (M)	Rmax (RU)
1:1 binding	MC1R	OP	-	-	-	-	-
1:1 binding	MC1R	OL	3.21	12.05	0.002320	$1.925\times 10^{-4}$	39.73
1:1 binding	MC1R	OC	17.1	$3.561\times 10^{5}$	0.001252	$3.515\times 10^{-9}$	7.676

## Data Availability

The supporting data of this article can be found within the paper. The microarray data have been deposited in the NCBI GEO database. Expression data from 5 μM of OL and OC-treated B16F10 cells. Available online: https://www.ncbi.nlm.nih.gov/geo/query/acc.cgi?acc=GSE237508 (accessed on 4 April 2024).

## Supplementary Materials

The following supporting information can be downloaded at: https://www.mdpi.com/article/10.3390/ijms25084479/s1.
