# Supplementary material for: Comparative Analysis of Olive-Derived Phenolic Compounds’ Pro-Melanogenesis Effects on B16F10 Cells and Epidermal Human Melanocytes
Source: Int J Mol Sci. 2024 Apr 19;25(8):4479. doi: 10.3390/ijms25084479 (PMC11050296; doi:10.3390/ijms25084479)
Supplement: Supplementary file 1 [file ijms-25-04479-s001.zip › ijms-2865502-supplementary.pdf]

Measuring Cytotoxicity of the Olive-derived Secoiridoids on B16F10 Cells

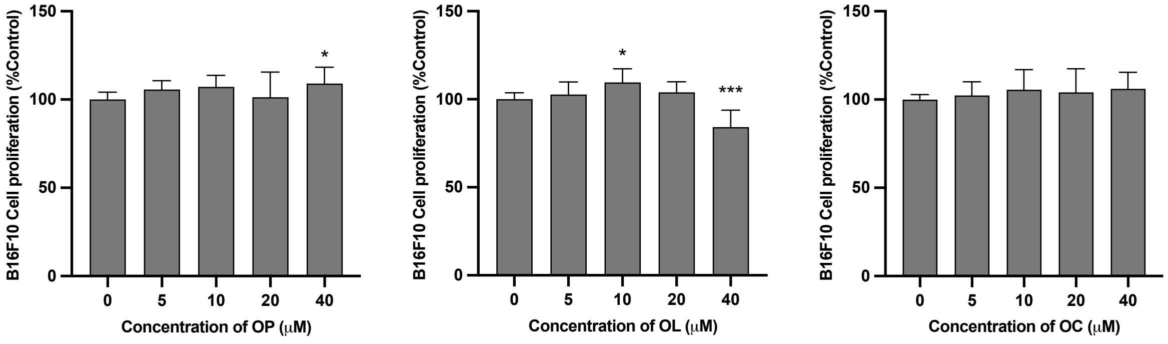

(a) (b) (c)

Figure S1. Cytotoxic effects of Oleuropein (OP), Oleocanthal (OL), and Oleacein (OC) were evaluated in B16F10 cells. Cells were treated with various concentrations of OP, OL, and OC for 48 hours. Each bar represents cell proliferation of (a) OP, (b) OL, and (c) OC.
